# A comprehensive analysis of radiosensitization targets; functional inhibition of DNA methyltransferase 3B radiosensitizes by disrupting DNA damage regulation

**DOI:** 10.1038/srep18231

**Published:** 2015-12-15

**Authors:** Hiroaki Fujimori, Akira Sato, Sota Kikuhara, Junhui Wang, Takahisa Hirai, Yuka Sasaki, Yasufumi Murakami, Ryuichi Okayasu, Mitsuko Masutani

**Affiliations:** 1 Division of Genome Stability Research, National Cancer Center Research Institute, 5-1-1 Tsukiji, Chuo-ku, Tokyo 104-0045, Japan; 2 Division of Chemotherapy and Translational Research, National Cancer Center Research Institute, 5-1-1 Tsukiji, Chuo-ku, Tokyo 104-0045, Japan; 3 Department of Biological Science and Technology, Faculty of Industrial Science and Technology, Tokyo University of Science, 2641 Yamazaki, Noda, Chiba 278-8510, Japan; 4 Department of Molecular Genetics, Medical Research Institute, Tokyo Medical and Dental University, 1-5-45 Yushima, Bunkyo-ku, Tokyo 103-8501, Japan; 5 Department of Radiation Oncology, Juntendo University Faculty of Medicine, 2-1-1 Hongo, Bunkyo-ku, Tokyo 113-8421, Japan; 6 Open Laboratory/Research Center for Radiation Protection, National Institute of Radiological Sciences, 4-9-1 Anagawa, Inage, Chiba 263-8555, Japan; 7 Department of Frontier Life Sciences, Nagasaki University Graduate School of Biomedical Sciences, 1-7-1, Sakamoto, Nagasaki 852-8588, Japan

## Abstract

A comprehensive genome-wide screen of radiosensitization targets in HeLa cells was performed using a shRNA-library/functional cluster analysis and DNMT3B was identified as a candidate target. *DNMT3B* RNAi increased the sensitivity of HeLa, A549 and HCT116 cells to both γ-irradiation and carbon-ion beam irradiation. *DNMT3B* RNAi reduced the activation of DNA damage responses induced by γ-irradiation, including HP1β-, γH2AX- and Rad51-foci formation. *DNMT3B* RNAi impaired damage-dependent H2AX accumulation and showed a reduced level of γH2AX induction after γ-irradiation. DNMT3B interacted with HP1β in non-irradiated conditions, whereas irradiation abrogated the DNMT3B/HP1β complex but induced interaction between DNMT3B and H2AX. Consistent with radiosensitization, *TP63*, *BAX*, *PUMA* and *NOXA* expression was induced after γ-irradiation in *DNMT3B* knockdown cells. Together with the observation that *H2AX* overexpression canceled radiosensitization by *DNMT3B* RNAi, these results suggest that *DNMT3B* RNAi induced radiosensitization through impairment of damage-dependent HP1β foci formation and efficient γH2AX-induction mechanisms including H2AX accumulation. Enhanced radiosensitivity by *DNMT3B* RNAi was also observed in a tumor xenograft model. Taken together, the current study implies that comprehensive screening accompanied by a cluster analysis enabled the identification of radiosensitization targets. Downregulation of DNMT3B, one of the targets identified using this method, radiosensitizes cancer cells by disturbing multiple DNA damage responses.

Biological radiosensitizers that are highly selective for cancer cells and display minimal toxicity to normal cells will greatly contribute to effective cancer radiotherapy^1^. Various types of radiosensitizers have been developed to date, including inhibitors of DNA repair enzymes such as DNA-dependent protein kinase^2^, poly(ADP-ribose) polymerase (PARP)^3^, poly(ADP-ribose) glycohydrolase^4^, and inhibitors of cell cycle checkpoint proteins such as checkpoint kinase 1^5^, heat-shock protein 90^6^, ataxia telangiectasia mutated kinase^7^, and histone deacetylase^8^. In addition, inhibitors of signaling pathway proteins such as RAS^9^, ErbB receptor tyrosine kinase^10^, and HER2^11^,^12^ have also been described as radiosensitizers. Bevacizumab is a radiosensitizer that blocks vascular endothelial growth factor (VEGF) in the tumor micro-environment^13^. Although some of these radiosensitizers have been evaluated in preclinical tests or clinical trials, their effectiveness is still limited.

Because cancer cells are heterogeneous and possess a diverse range of mutations and alterations, suitable radiosensitization targets may differ among individual cancer types; therefore, a comprehensive understanding of the mechanisms of radiosensitization will aid the identification of suitable target proteins. To our knowledge, comprehensive RNAi screening for inducing radioresistancy was previously reported by using p53 proficient cancer cells, U2OS^14^. However, inactivation of p53 is by far the most common alteration across all forms of cancer. Therefore this study aims to comprehensively identify genes that promote the radiosensitization of p53-inactivated cancer cells, HeLa when downregulated. Our negative screening following a functional cluster analysis identified the *de novo* DNA methyltransferase (DNMT) 3B as a candidate.

DNA methyltransferases are thought to be involved not only in epigenetic regulation but also in DNA repair systems. DNMT1 deficient cells show activation of the ATR pathway accompanying γ-H2AX, CHK1/2 phosphorylation. Overexpression of mutant DNMT1 defective in DNA methylation activity rescued the activation of DNA damage response in *DNMT1* deficient cells^15^, suggesting that DNMT1 could contribute to DNA double strand break (DSB) repair in a DNA methylation independent manner. In this study, we demonstrated that DNMT3B regulates HP1β and H2AX to protect cells from ionizing radiation (IR). We showed that *DNMT3B* knockdown induces radiosensitization in *DNMT3B* expressing cancer cell lines, and in a xenograft model. As a mechanism, DNMT3B dysfunction impaired HP1β foci-formation and H2AX accumulation induced by IR. *DNMT3B* knockdown HeLa cells showed similar phenotypes to H2AX deficient cells after IR, including low survival ratio and impairment of G1/S arrest. Furthermore, *DNMT3B* RNAi dependent radiosensitization was rescued by *H2AX* overexpression. Together with the detection of interaction between DNMT3B and H2AX induced by IR, our current study suggested that DNMT3B regulates IR-induced HP1β foci-formation and H2AX accumulation, and consequent DNA damage responses thereby protecting cells from cell death.

DNMT3B is overexpressed in various cancer cells and *DNMT3B* overexpression is reported as a poor prognostic factor in patients^16^. Although combination of radiotherapy and DNMT inhibitors^17^ has been reported, the correlation between *DNMT3B* overexpression and the resistance to radiotherapy has not been extensively studied.

Our current study supports the notion that DNMT3B is a potential target for inducing radiosensitization. Furthermore, comprehensive screening accompanied by cluster analysis might be useful for the identification and evaluation of radiosensitization targets.

## Materials and Methods

### Cell culture

HeLa (ATCC), T-REx HeLa (Invitrogen) and HCT116 cells (ATCC) were cultured in Minimum Essential Medium (Sigma) supplemented with 10% FBS, 1% penicillin-streptomycin (Invitrogen), and non-essential amino acids (Invitrogen). A549 (ATCC) cells were cultured in RPMI-1640 medium containing 10% FBS, 1% penicillin-streptomycin, and non-essential amino acids. For S phase synchronization, HeLa cells were treated twice with 2 mM thymidine (Sigma) for 16 h.

### Gene expression analysis

Quantitative reverse transcription PCR was performed using the CFX96 real-time PCR system (Bio-Rad) and SYBR Select Master Mix (Invitrogen), as described previously^18^. The sequences of the PCR primers are listed in Supplementary Table S1.

### Vector construction

Expression vectors for short hairpin RNAs (shRNAs) were constructed using the BLOCK-iT™ Pol II miR RNAi Expression Vector Kit with EmGFP (Invitrogen). Briefly, RNAi sequences for human *DNMT3B*, designed using the BLOCK-iT™ RNAi Designer program (Invitrogen), were annealed and ligated with linearized pcDNA6.2™-GW/EmGFP-miR plasmid. The efficiency of knockdown of the target gene was examined by quantitative reverse transcription PCR (Bio-Rad). The shRNA sequences are listed in Supplementary Table S2.

Overexpression vectors for human H2AX and DNMT3B were constructed using pIRES-hrGFP (Promega). Briefly, *H2AX* and *DNMT3B* ORF sequences were amplified from a HsCD00437738 clone and a HsCD00082655 clone (DNASU) by PCR, respectively. These sequences were inserted into the pIRES-hrGFP vector as an in-frame fusion to the FLAG-epitope tag encoded by the vector. The sequences of the PCR primers are listed in Supplementary Table S1.

### Vector transfection

RNAi (RNA interference) or overexpression vectors were transfected into indicated cancer cell lines using Lipofectamine LTX reagent (Invitrogen). Mock vectors were used for negative control. Briefly, 4 × 10^4^ cells were seeded into a 24-well plate (Thermo Scientific) and transfected with 0.25 μg of DNA at 24 h and 48 h post-seeding. The efficiency of transfection was examined using the fluorescence-activated cell sorting technique for EmGFP, as described previously^19^.

### Negative selection using a shRNA library

Negative screening was performed using the Decode RNAi Pooled Lentiviral shRNA Screening Libraries: Annotated Genome Negative Selection Kit (Thermo Scientific). Briefly, T-REx HeLa cells (Invitrogen) were infected with a lentiviral siRNA expression library (Thermo Scientific) using TransDux reagent (System Biosciences). Green fluorescent protein-positive cells were selected by puromycin treatment and divided into two populations, one of which was irradiated with 0.75 Gy using a ^137^Cs irradiator (Gy/min, Best Theratronics); the other served as a non-irradiated control. Four days after irradiation, genomic DNA was purified from each population using a DNA purification kit (Dojindo). Barcode sequences were amplified from 0.5 μg of genomic DNA, purified by gel extraction, and labeled using a Genomic DNA Enzymatic Labeling Kit (Agilent Technologies). After purification using Amicon Ultra-0.5 ml centrifugal filters (Millipore), the labeled barcode sequences were hybridized with microarray slides for 17 h and the slides were then washed according to the Agilent CpG microarray protocol. Cluster analysis of the extracted genes was performed using the information in the GeneCards database (http://www.genecards.org/) and KEGG (http://www.genome.jp/kegg/).

### Colony formation assay

A clonogenic survival assay was performed as described previously, with some modifications^20^. Briefly, 20 h prior to irradiation, cells were seeded into 6-well plates in triplicate (Thermo Scientific). Seven days after irradiation, the colonies were fixed with 4% neutralized formalin and stained with 0.02% crystal violet. Colonies comprising more than approximately 50 cells were counted.

### Antibodies, immunostaining and western blotting

Western blotting and immunostaining were performed as described previously^21^. Briefly, for immunostaining, cells were fixed with neutralized formalin and then treated with Block Ace blocking solution (DS Pharma Biomedical) containing 0.1% Triton X-100 (Wako). The cells were then incubated with primary antibody followed by fluorescent secondary antibodies diluted in blocking solution. For western blotting, whole cells were washed with cold PBS and extracts were prepared using Nu-PAGE lysis buffer (Invitrogen). The chromatin fractions were isolated by centrifuging the extracts at 15,000 *g* for 20 min following treatment with RIPA buffer (50 mM Tris-HCl (pH 8.0), 150 mM NaCl, 0.5% NP-40, 0.5% sodium deoxycholate, and 0.1% SDS). Proteins were transferred onto a polyvinylidene difluoride membrane using the iBlot system (Invitrogen) and probed with the appropriate primary antibodies. The membrane was then incubated with corresponding horseradish peroxidase-conjugated secondary antibodies and the antigen-antibody complexes were visualized using Immobilon Western Chemiluminescent HRP Substrate (Millipore). Details of the antibodies used are provided in Supplementary Table S3.

### Transfection of siRNAs

Cells were transfected with a Silencer Select Validated siRNA targeting *DNMT3B* (s4221; Invitrogen) using Lipofectamine RNAi MAX reagent (Invitrogen), according to the manufacturer’s protocol. Briefly, prior to transfection, 3 × 10^4^ cells were seeded into 24-well plates (Thermo Scientific) in growth medium without antibiotics. A solution comprising 1 μl of Lipofectamine RNAi MAX and 5 pmol of siRNA in 100 μl of Opti-MEM (Invitrogen) was added to each well. AllStars Negative Control siRNA (Qiagen) was used as a control.

### Cell growth analysis

Cellular proliferation was analyzed by using CCK assay kits (cell counting kit-8, Dojindo Laboratories), according to the manufacturer’s protocol. Briefly, 10,000 HeLa cells were seeded in 12-well plates and vector/siRNA were transfected. These cells were seeded in 96-well plate 24 h after transfection and irradiated 48 h after transfection. The absorbance at 450/600 nm was measured 3 days after irradiation.

### Protein interaction detection

Detection of protein direct interaction was analyzed by using Duolink *in situ* PLA (Proximity Ligation Assay) technologies (Sigma), according to the manufacturer’s protocol. Briefly, 10,000 HeLa cells were seeded in 12-well plates and DNMT3B-Flag overexpression vector was transfected. Forty-eight hours after transfection, these cells were seeded in 8-well-chamber slides (Thermo Scientific) and irradiated 72 h after transfection. These cells were fixed with formalin 1 h after irradiation. Details of the antibodies used are provided in Supplementary Table S3.

### Cell cycle analysis

Flow cytometric analyses were performed as described previously^22^. Briefly, cells were fixed with 70% ethanol, stained with PBS containing 50 μg/ml propidium iodide and 20 μg/ml RNase A (Sigma), and then analyzed using a Sony EC800 flow cytometer. Data acquisition was performed using EC800 analyzer software (Sony).

### Animal experiments

Cell implantation was performed as described previously^19^, with some modifications. Briefly, A549 cells were transfected with the *DNMT3B*-specific or negative control siRNA 1 day before implantation. The following day, 8 × 10^5^ transfected cells were suspended in 50 μl of Growth Factor Reduced Matrigel (BD Biosciences) and injected subcutaneously into both legs of 6-week-old male Balb/c nude mice (Charles River Laboratories). Four mice were injected with each siRNA. On days 2 to 4 after implantation, the mice were subjected to daily irradiation (4 Gy) of the right leg; irradiation was restricted to the right leg only by protecting the rest of the body with a lead shield. Tumor volumes were observed once a week and mice were euthanized 37 days after implantation. Tumor volumes were measured with micrometer calipers and calculated as follows: volume = pi4/3 × (smallest diameter) × (largest diameter) × (height). All animal studies were approved by Animal Experimental Committee of National Cancer Center and were performed in accordance with the Guidelines for Animal Experiments of the National Cancer Center, which meet the ethical guidelines for experimental animals in Japan.

## Results

### A comprehensive analysis to identify radiosensitization targets

**Figure 1:**
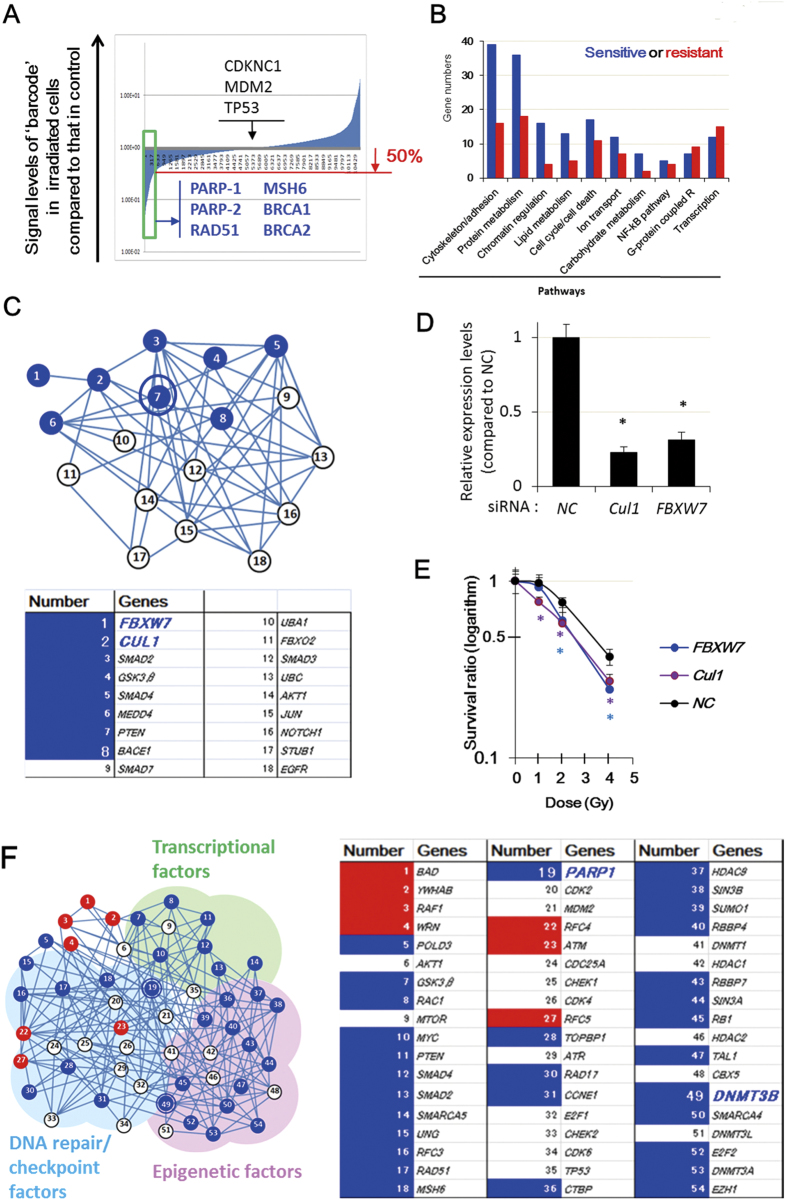
Comprehensive analysis identified radiosensitizing targets including *DNMT3B*. (**A and B**) The results of a comprehensive negative screening analysis of radiosensitivity-related genes in T-REx HeLa cells transfected with a shRNA library. (**C**) Analysis of a functional network cluster around the genes encoding *phosphatase and tensin homolog* (*PTEN*) and *glycogen synthase kinase 3*β (*GSK3*β) (upper panel). The numbers shown in blue indicate genes whose knockdown induced radiosensitization (lower panel). (**D**) Confirmation of RNAi-mediated knockdown of *CUL1* (a) and *FBXW7* (b) expression in T-REx HeLa cells. Error bars: SE. Asterisks show *P* < 0.05. (**E**) Radiosensitization induced by *CUL1* or *FBXW7* knockdown in T-REx HeLa cells analyzed by colony formation assay. Mean + SE. Asterisks show *P* < 0.05. (**F**) Cluster analysis and validation of novel candidate genes. A functional network cluster of the target genes around PARP-1 (left panel). The targets whose downregulation induced radiosensitization and radioresistance are shown in blue and red, respectively (right panel).

To identify novel targets for radiosensitization, negative screening was performed by transfecting T-REx HeLa cells with a lentiviral shRNA library containing approximately 10,000 genes. To identify genes whose knockdown causes radiosensitivity from low doses of γ–irradiation, two populations of cells were either non-irradiated or irradiated at 0.75 Gy and cultured for 4 days. Genomic DNA was extracted from each cell population and hybridized to microarray slides. PCR amplification of specific barcode sequences following microarray hybridization revealed differences in the abundances of each shRNA clone between non-irradiated and irradiated cells. Using this system, irradiation-induced depletion of cells containing a specific shRNA indicated that knockdown of the corresponding target gene caused radiosensitivity. The 164 genes showed at least 2-fold radiosensitizing effects and knockdown of 91 genes gave at least 2-fold radioresistant effects (Fig. 1A); this list included *PARP-1*, *PARP-2*, and *RAD51*, knockdown of which induced radiosensitization in previous studies^23^,^24^. On the other hand, as reported previously, knockdown of the genes encoding cyclin-dependent kinase inhibitor 1C, tumor protein P53, and MDM2 proto-oncogene E3 ubiquitin protein ligase, which are inactive in HeLa cells^25^, did not affect radiosensitivity (Fig. 1A). Taken together, these results confirmed that the negative screening system worked as expected.

**Figure 2:**
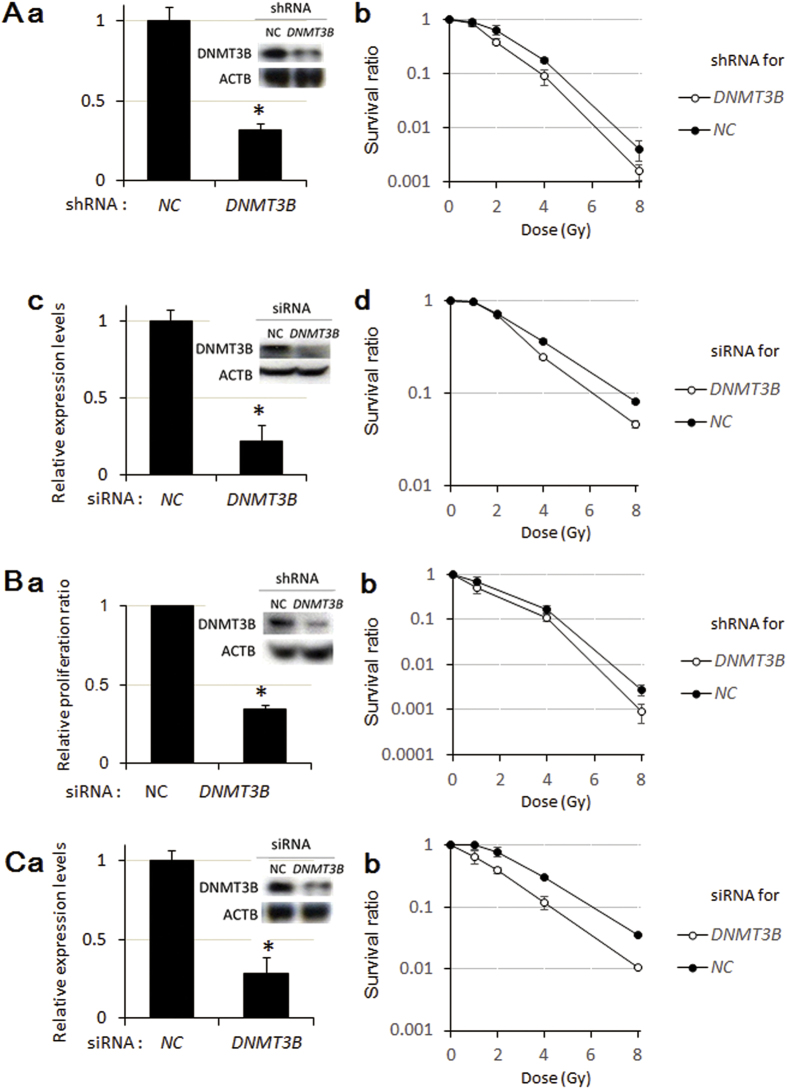
Radiosensitization in *DNMT3B* knockdown cells. (**A**) The *DNMT3B* knockdown increased radiosensitivity of HeLa cells. The cells were treated with shRNA of *DNMT3B* and the mRNA level and protein level of *DNMT3B* was detected by qRT-PCR (a) and western blot (a, inserted), respectively. The effect of *DNMT3B* knockdown with shRNA on the radiosensitivity of HeLa cells was analyzed by colony formation assay (b). The enhancement ratio at 10% survival was 1.2 (c-e). The knockdown levels of *DNMT3B* after transfection of *DNMT3B* specific siRNA at the mRNA (c) and protein (c, inserted) levels. The effect of *DNMT3B* knockdown with siRNA on the radiosensitivity of HeLa cells was analyzed by colony formation assay (e). The enhancement ratio at 10% survival was 1.3. (**B** and **C**) *DNMT3B* RNAi radiosensitized in A549 (**B**) and HCT116 (**C**) cell lines. The mRNA and protein levels of *DNMT3B* in A549 transfected with shRNA for *DNMT3B* (Ba with an inserted panel) and HCT116 transfected siRNA for *DNMT3B* (Ca with an inserted panel). Error bars: SE. The colony formation analysis was carried out (Bb and Cb). Error bars: SE. The enhancement ratio at 10% survival was 1.2 for A549 (Bb) and 1.4 for HCT116 (Cb). Asterisks show *P* < 0.05. The mRNA level of *DNMT3B* was normalized with that of *GUSB* (Aa, Ac, Ba and Ca).

Figure 1B shows the classification of the 157 genes in cellular pathways, whose knockdown can induce either radiosensitization or radioresistance. Notably, the list of candidate genes contained not only DNA repair-related genes, but also epigenetic regulators, inflammation-related genes, and genes with other functions (Fig. 1B). To identify suitable targets for radiosensitization and exclude false positives, we attempted a cluster analysis of the candidate genes by using the information in the GeneCards database (http://www.genecards.org/) and KEGG (http://www.genome.jp/kegg/), which are related to the reported functions and interactions of each gene. By combining this information and the results of screening, we identified a small cluster of ‘hit-genes’ including phosphatase and tensin homolog (PTEN) and glycogen synthase kinase 3β (GSK-3β) (Fig. 1C), which have both been reported as radiosensitization targets in some genetic backgrounds^26^,^27^. RNAi-mediated knockdown of two genes included in this cluster, namely, cullin 1 (*CUL1*) and F-box and WD repeat domain containing 7, E3 ubiquitin protein ligase (*FBXW7*), promoted radiosensitization of T-REx HeLa cells to γ-irradiation (Fig. 1D and E). Downregulation of the seven candidates, which did not constitute clusters, did not show radiosensitization (data not shown). On the other hand, knockdown of each candidate that was present in clusters (four genes including *CUL1*, *DNMT3B* and *FBXW7*) respectively caused radiosensitization in T-REx HeLa cells or HeLa cells (Fig. 1E and 2, and data not shown), suggesting that the false positive rate of the screening system becomes low when cluster analysis is combined. Taken together, these results suggest that cluster analyses are useful for excluding noise and identifying true positive targets.

Previous studies showed that inhibition of PARP induces sensitization to several types of radiotherapy^20^,^28^; therefore, the results of the comprehensive negative screening were analyzed from the view point of PARP-associated genes. A large cluster of hit-genes around *PARP-1* was identified; consistent with the known role of PARP-1, this cluster included genes associated with cell cycle/DNA repair, transcription, and epigenomic regulation (Fig. 1F). Furthermore, the cluster included known radiosensitizing genes, including those encoding *mutS homolog 6*, *RAD17*, and *RAD51*, suggesting that the hit-genes in this cluster may be suitable targets for radiosensitization.

In the subsequent analyses, we focused on the candidate gene *DNMT3B* in this cluster because its expression level is often increased in various types of cancers^29^. Furthermore, although stem cells express DNMT3^29^, the expression of this protein is generally restricted to minor organs such as testes and spleen, suggesting that it may be a more suitable target for radiosensitization than ubiquitously expressed DNA repair proteins and transcription factors.

### Radiosensitization in *DNMT3B* knockdown cells

To determine whether dysfunction of DNMT3B induces radiosensitization, we compared the survival rates of *DNMT3B*-knockdown and control HeLa cells exposed to γ-irradiation. In these experiments, an RNAi expression vector constructed using a commercially available kit (BLOCK-iT™ Pol II miR RNAi Expression Vector Kit with EmGFP) (Fig. 2Aa) and a specific siRNA (Fig. 2Ac and d) were used to knockdown *DNMT3B*. RNAi of *DNMT3B* caused increased sensitivity of HeLa cells to both γ-irradiation and carbon-ion beam irradiation (Fig. 2Ab and e and Fig. Supplemental (S)1). Additionally, we examined whether DNMT3B dysfunction induced radiosensitization in other cancer cell lines exhibiting high DNMT3B expression. Knockdown of *DNMT3B* enhanced the radiosensitivity of lung cancer A549 cells to both γ-irradiation and carbon-ion beam irradiation (Fig. 2Ba-b and Fig. S1). Furthermore, knockdown of *DNMT3B* enhanced the radiosensitivity of colon cancer HCT116 cells to both γ-irradiation and carbon-ion beam irradiation (Fig. 2Bc-d and Fig. S1). Together, these results suggested that DNMT3B dysfunction induced radiosensitization in cancer cell lines exhibiting high expression of DNMT3B.

### The formation of γ-irradiation-induced HP1β foci is impaired in *DNMT3B* knockdown cells

**Figure 3:**
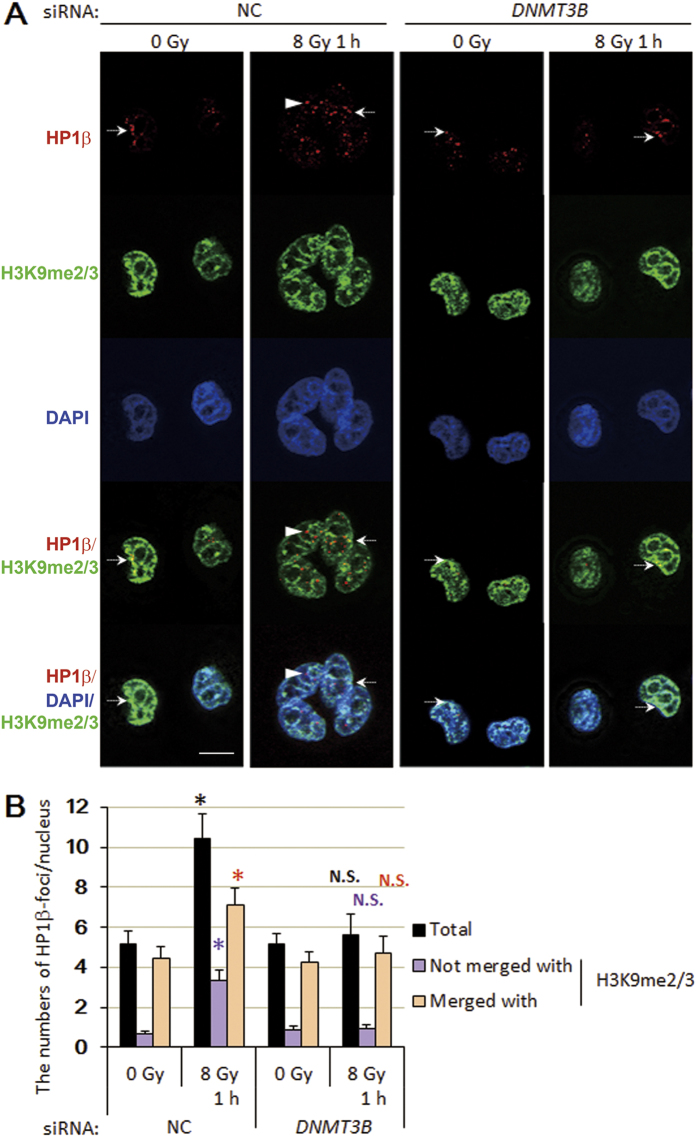
Knockdown of *DNMT3B* disrupts γ-irradiation-induced formation of HP1β foci. (A and B) Formation of HP1β foci in control and *DNMT3B* knockdown cells 1 h after irradiation (8 Gy). (**A**) Immunostaining data. Arrows: HP1β foci merged with H3K9me2/3. Arrows heads: HP1β foci not merged with H3K9me2/3. (**B**) Quantitative data. HP1β foci were counted at least in 50 cells. Significant differences between the number of foci in non-irradiated and irradiated cells were determined. Error bars: SE. Asterisks show *P* < 0.05. Scale bar: 10 μm. N.S.: *P* ≧ 0.05.

We chose HeLa cells that moderately express DNMT3B to explore the mechanism of radiosensitization by *DNMT3B* knockdown, because DNMT3B knockdown itself induces substantial growth suppression in tumor cells, if *DNMT3B* is overexpressed^30^. First, we examined the changes of expression levels of DNA damage-response factors. Although a major role of the DNMT family is regulating promoter activities through 5-methylcytosine modification, basal expression levels of repair and cell-cycle checkpoint factors were not reduced or increased more than 2 fold in *DNMT3B* knockdown HeLa cells (Fig. S2), therefore we considered that knockdown of *DNMT3B* may mainly contribute to DNA repair machinery in a more direct manner. Previous studies showed that DNMT3B interacts with HP1 and the accumulation of HP1β at DSB sites has been reported as an early response to irradiation^31^,^32^; therefore, we examined whether knockdown of *DNMT3B* impaired the regulation of HP1β in HeLa cells after γ-irradiation. Consistent with the previous reports, γ-irradiation induced the formation of HP1β foci in control cells and this process was independent of the presence or absence of the H3K9me2/3 modification (Fig. 3A and B). Of note, irradiation-induced formation of HP1β foci was largely impaired in *DNMT3B* knockdown cells (Fig. 3A and B). It is reported that loss of heterochromatin formation in HP1β knockdown cells is associated with increased radiosensitivity^33^, and HP1β regulates DNA repair and cell cycle arrest after γ-irradiation^31^. The results thus suggest that radiosensitization caused by knockdown of DNMT3B involves defective HP1β functioning.

### H2AX signaling is disturbed and delayed when DNMT3B is dysfunctional

**Figure 4:**
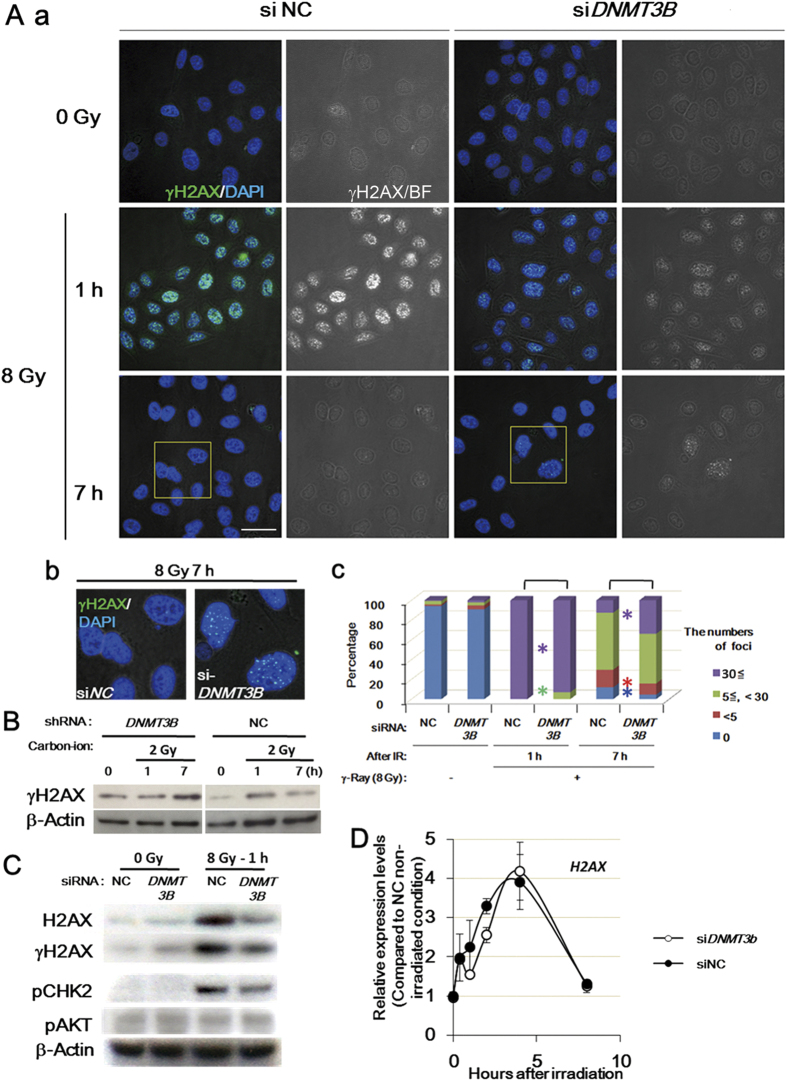
Knockdown of *DNMT3B* reduces the formation of γH2AX foci after irradiation. (**A and B**) The formation of γH2AX foci 1 h and 7 h after exposure of control and *DNMT3B* knockdown cells to γ-irradiation (**A**) or carbon-ion beam irradiation (**B**). Failure of quick and robust γH2AX induction after γ-irradiation in *DNMT3B* knockdown cells (Aa); One hour after γ-irradiation, *DNMT3B* knockdown cells exhibited γH2AX foci with low intensity compared to that in NC cells. On the other hand, 7 h after irradiation, γH2AX signals in *DNMT3B* knockdown cells became more intensive compared to NC cells (Ab and Ac). Green square regions in Aa were enlarged in Ab. The γH2AX foci were counted at least in 140 cells, respectively. Asterisks show *P* < 0.05 (X2-test). Western blot analysis showed that carbon-ion beam irradiation also failed to the quick activation of γH2AX in *DNMT3B* knockdown cells (**B**). (**C**) H2AX protein levels in control and *DNMT3B* knockdown cells exposed to γ-irradiation. Scale bar: 20 μm. (**D**) *H2AX* mRNA levels in control and *DNMT3B* knockdown cells after 8 Gy γ-irradiation. The expression level of *H2AX* was normalized to that of *GUSB*. Error bars: SE.

To investigate the effect of knockdown of *DNMT3B* on the activation of the repair/checkpoint pathway, we focused on H2AX, which is phosphorylated in response to DNA double-strand breaks (DSBs) to form γH2AX, and this process was partly modulated by HP1β foci-formation^34^. At 1 h post-irradiation, there was only a slight reduction of the numbers of γH2AX foci in *DNMT3B* knockdown cells than in the control (Fig. 4A), however, the intensity of the γH2AX signal was substantially weaker in the *DNMT3B* knockdown cells than in the control cells (Fig. 4A). In contrast, at 7 h post-irradiation, both the signal intensity and number of γH2AX foci were higher in the *DNMT3B* knockdown cells than in the controls (Fig. 4A). Western blot analysis revealed a similar delay in the induction of γH2AX foci when the *DNMT3B* knockdown cells were exposed to carbon-ion beam irradiation (Fig. 4B). These results indicate that the acute phosphorylation of H2AX is impaired substantially and following DSB repair process may be disturbed when *DNMT3B* is dysfunctional.

It was reported previously that accumulation of the H2AX protein is induced after DNA damage^35^, therefore, we examined whether the accumulation is impaired under *DNMT3B* knockdown conditions. As expected, the H2AX protein was accumulated in the control cells 1 h after γ-irradiation, and this accumulation was decreased markedly by knockdown of *DNMT3B* (Fig. 4C). In contrast, irradiation-dependent induction of the *H2AX* mRNA was not significantly affected by knockdown of *DNMT3B* (Fig. 4D). The reduced accumulation of H2AX protein could have contributed to the relatively low signal-intensity and the lower number of γH2AX foci in *DNMT3B* cells versus control cells. Exposure to γ-irradiation also induced phosphorylation of CHK2 and RAD51 foci, and these inductions were attenuated by knockdown of *DNMT3B* (Fig. 4C and S3) suggesting that knockdown of *DNMT3B* disrupts the DSB-induced signaling cascade. Taken together, these results implied that knockdown of *DNMT3B* attenuates H2AX accumulation, γH2AX formation, and downstream process.

### DNMT3B interacts with H2AX after irradiation, contributing to cell survival after irradiation

**Figure 5:**
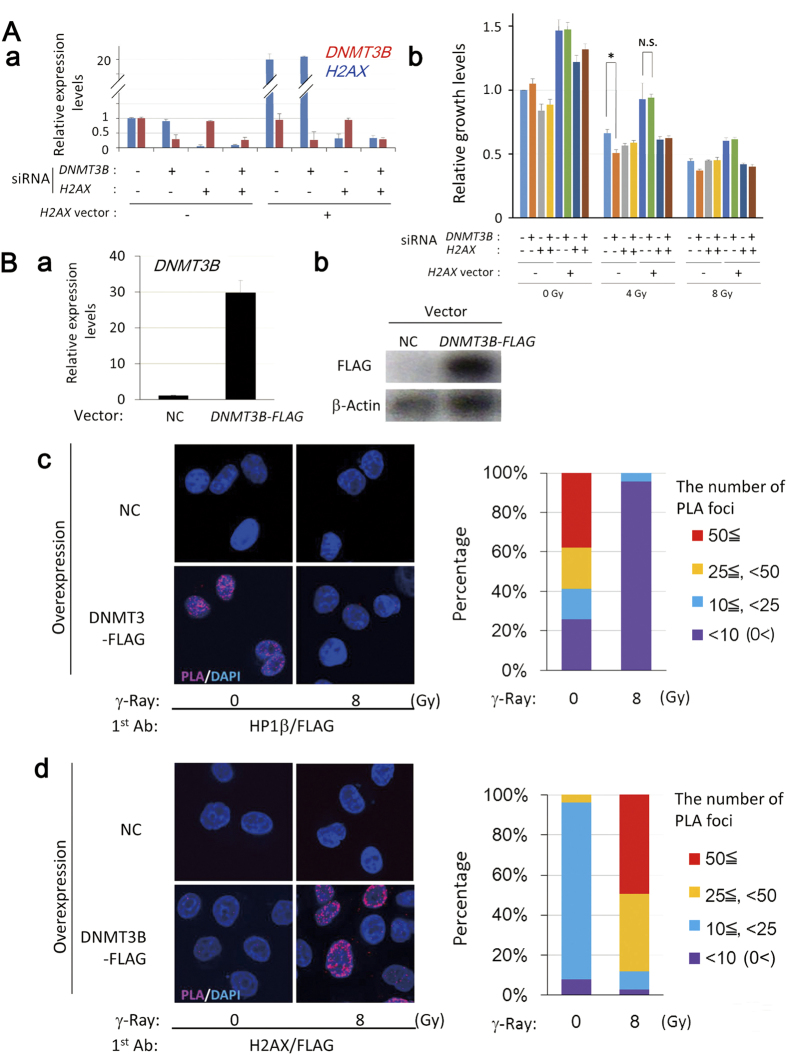
Knockdown of *DNMT3B* radiosensitizes through impairment of H2AX regulation after DNA damage. (**A**) *H2AX* overexpression suppressed radiosensitization induced by *DNMT3B* RNAi in HeLa cells. MTT assay was performed 4 days after γ-irradiation. Relative mRNA expression levels of *DNMT3B* and *H2AX* in indicated conditions (a). The expression level was normalized to that of *GUSB*. Error bars: SE. Relative growth levels of HeLa cells in each transfected condition (b). Error bars: SE. (**B**) Interaction between DNMT3B and HP1β or H2AX observed 1 hr after 8 Gy irradiation. Confirmation of *DNMT3B-FLAG* overexpression at mRNA level (a) and protein level (b). The number of PLA foci between DNMT3B and HP1β was decreased after irradiation (c), whereas that of DNMT3B and H2AX was increased after irradiation (d). Scale bar: 10 μm.

To confirm that the impairment of damage-dependent H2AX accumulation contributes to radiosensitization induced by *DNMT3B* knockdown, we examined whether *H2AX* overexpression abrogates the radiosensitization effects by *DNMT3B* RNAi or not. By the transfection of H2AX vector, *H2AX* mRNA level showed approximately 20 fold increase and further treatment of *H2AX* RNAi downregulated the both overexpressed and endogenous *H2AX*. With this system, radiosensitization effect of *DNMT3B* knockdown was analyzed (Fig. 5Aa). Consistent with the results of the colony formation assay, DNMT3B RNAi decreased cell number after γ-irradiation in a growth assay, however in *H2AX* overexpressed cells, *DNMT3B* RNAi no longer induced radiosensitization (Fig. 5A). These results suggest that impairment of damage-dependent H2AX accumulation significantly contributes to radiosensitization in *DNMT3B* knockdown cells, and DNMT3B is required for γ-irradiation-induced H2AX accumulation. We also noted that overexpression of H2AX leads to growth enhancement and conferred radioresistant effects.

Our current study demonstrated that *DNMT3B* RNAi did not markedly alter basal expression levels of DNA damage associated genes but impaired HP1β foci-formation and H2AX accumulation after irradiation. This motivated us to investigate whether DNMT3B directly regulates these proteins. To test the interaction between DNMT3B and HP1β or H2AX, we overexpressed DNMT3B-FLAG in HeLa cells (Fig. 5Ba, b) to detect PLA foci formed between DNMT3B and HP1β or H2AX. We observed PLA foci formed between DNMT3B and HP1β in the absence of irradiation, however, the foci were lost one hour after 8 Gy irradiation, suggesting impaired DNMT3B/HP1β interaction (Fig. 5Bc). In contrast, PLA foci formed between DNMT3B and H2AX were observed only after γ-irradiation (Fig. 5Bd). These results suggest that DNMT3B may directly or indirectly interact with HP1β in close proximity in non-irradiated condition and H2AX after IR.

### Knockdown of *DNMT3B* impairs G1/early S arrest and activates the P53 downstream pathway after irradiation

**Figure 6:**
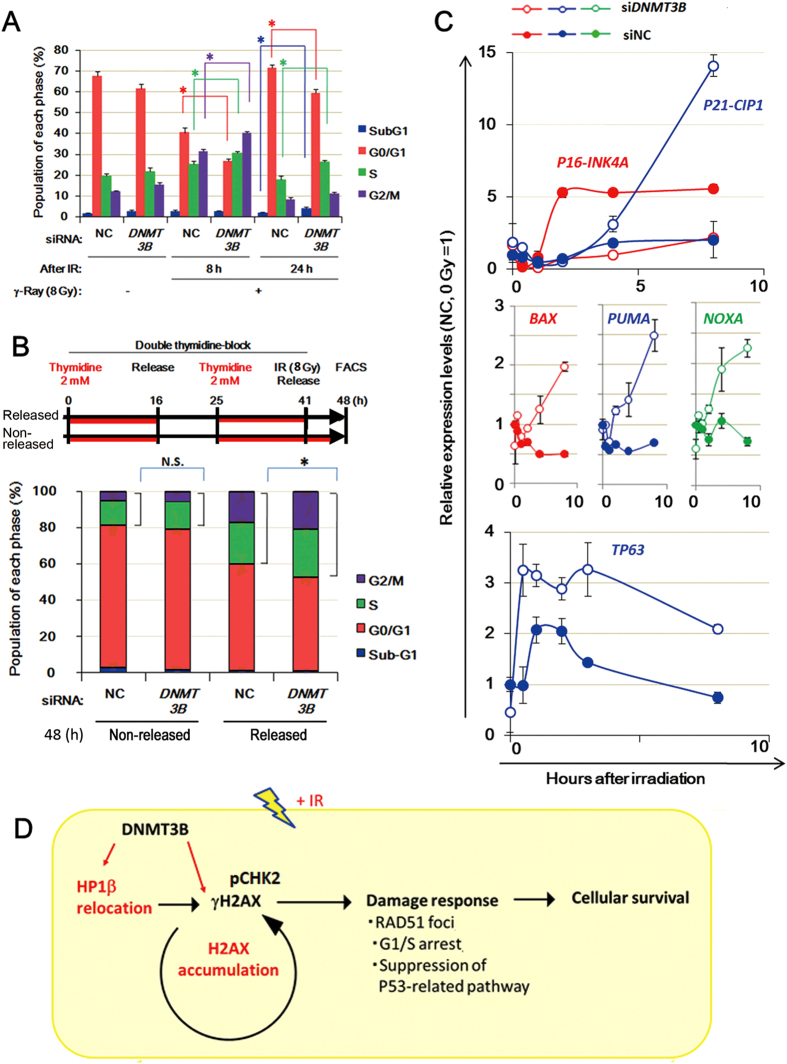
Knockdown of *DNMT3B* activates the P53-dependent pathway after γ-irradiation of HeLa cells. (**A**) The G1, G2/M and sub-G1 populations of *DNMT3B* knockdown cells 8 h and 24 h after irradiation (8 Gy). Error bars: SE. Asterisks show *P* < 0.05. (**B**) The G1, G2/M and sub-G1 populations of synchronized *DNMT3B* knockdown cells 7 h after irradiation. The upper panel shows the scheme of synchronization experiment. Error bars: SE. Asterisks show *P* < 0.05. (**C**) The activation of P53 pathway genes in irradiated *DNMT3B* knockdown cells. The expression level was normalized to that of *GUSB*. Error bars: SE. (**D**) A model for DNMT3B functions in IR response. DNMT3B interacts with the DSB sensor proteins, H2AX and HP1β and protects cells from irradiation damage through regulation of damage associated responses.

One phenotype of H2AX dysfunction is impairment of G1 and S phase arrest and defective DSB repair resulting in carrying over of DNA lesions into the G2/M phase after irradiation^36^. To confirm that knockdown of *DNMT3B* also attenuates the activation of cell cycle arrest machinery after γ-irradiation, we compared cell cycle patterns between the control and *DNMT3B* knockdown conditions after γ-irradiation in HeLa cells. Knockdown of *DNMT3B* slightly reduced the population in the G1 phase. Eight hours after irradiation, a further decrease in the G1 population and an increase in the S and G2/M population occurred in the *DNMT3B* knockdown cells (Fig. 6A), suggesting that knockdown of *DNMT3B* attenuates G1 arrest after irradiation. To clarify this process, we irradiated HeLa cells synchronized at the G1/S phase boundary and examined their progression into the S and G2/M phases after release (Fig. 6B, upper panel). Seven hours after irradiation of *DNMT3B* knockdown cells, the S-G2/M phases population was increased and the G1 population was decreased (Fig. 6B, lower panel). These results indicate that, when DNMT3B is dysfunctional the checkpoint system at the G1/early-S stage is impaired, resulting in arrest at the S and G2/M phases.

Additionally, we examined whether DNMT3B RNAi enhanced expression of cell-death related genes after γ-irradiation in HeLa cells. A marked time-dependent induction of *P21-CIP1* expression was observed in irradiated *DNMT3B* knockdown cells (Fig. 6C, top panel). Consistently, the expression levels of *BAX*, *NOXA* and *PUMA*, which are apoptosis-related components of the P53 downstream pathway, were induced in response to irradiation in *DNMT3B* knockdown cells but not in control cells (Fig. 6C, middle panel). Furthermore, expression of p63, another activator of the P53 downstream pathway, was strongly increased just after irradiation in DNMT3B knockdown cells, which could explain the lower survival of *DNMT3B* knockdown HeLa cells that exhibit ARF/P53 cascade inactivation (Fig. 6C, bottom left panel).

### *DNMT3B* dysfunction induces radiosensitization also in a mouse xenograft model

**Figure 7:**
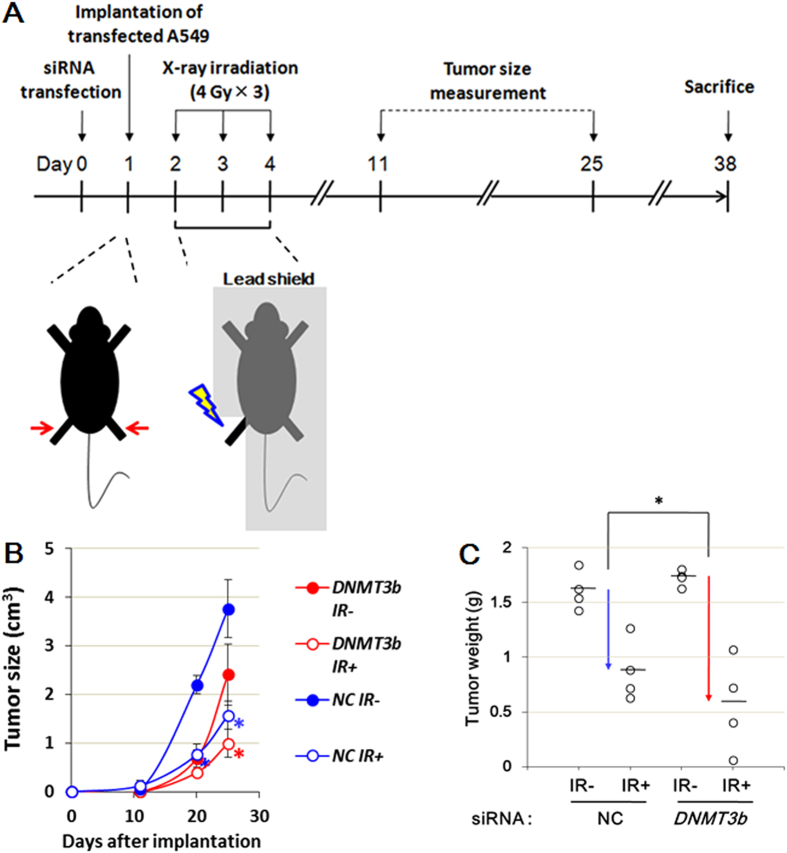
Knockdown of *DNMT3B* enhances radiosensitivity in tumor xenograft model. An overview of the xenograft experimental procedure (**A**). The temporal development of the tumor masses in animals injected with control and *DNMT3B* knockdown A549 cells (**B**). Error bars: SE. Asterisks show *P* < 0.05. The tumor weights at day 38 showing that knockdown of *DNMT3B* increased the susceptibility of the tumors to γ-irradiation. Bars show the mean tumor weight. Asterisks show *P* < 0.05 (**C**).

Finally, a xenograft model was also employed to examine whether knockdown of *DNMT3B* suppresses the growth of irradiated A549 tumors (Fig. 7A,B). Mice were subcutaneously injected with A549 cells transfected with either the control or *DNMT3B*-specific siRNA, and then γ-irradiated on days 2 to 4 after implantation (Fig. 7Ba and b). The knockdown of *DNMT3B* itself slightly suppressed the tumor growth in the absence and further reduced the tumor growth after γ-irradiation (Fig. 7Bc and d), confirming the radiosensitization effect of *DNMT3B* knockdown in the xenograft model.

## Discussion

Targeting or inhibition of single genes is the typical approach to modulating biological radiosensitivity; however, this process has been ineffective, even in cell-based studies and also in the xenograft models or in clinical studies, possibly due to differences in gene abnormalities among individual cancer cells. For this reason, it is necessary to examine suitable radiosensitization targets in a genome-wide manner. Because p53 pathway is widely inactivated in various types of cancer cells, we focused on the radiosensitization targets, which are effective in p53 defective cells. Here, a comprehensive screen of a shRNA library in T-Rex HeLa cells identified around 150 candidate radiosensitization-related genes, which were classified into various functional groups. Notably, we showed that the generation of gene clusters based on networks of functional interactions is a useful method of identifying genes of interest. This comprehensive analysis method enabled the identification of radiosensitizing clusters of genes rather than single genes. In addition, knowledge of genes whose inhibition causes radioresistance may also aid the selection of optimal radiosensitization targets and enhance current understanding of the mechanisms of radioresistance in cancer cells.

Based on the results of a comprehensive screen, downregulation of the seven candidates selected without the cluster analysis did not promote radiosensitization, but downregulation of the four candidates identified through the cluster analysis promoted radiosensitization of T-REx HeLa cells or HeLa cells, suggesting that the identification of false positive targets can be reduced by combining screening with a functional cluster analysis.

We focused on the epigenetic regulator DNMT3B, which was identified as a candidate target gene in the cluster of DNA repair/epigenetic factors/transcriptional factors, because it is overexpressed frequently in cancers. Knockdown of *DNMT3B* enhanced the radiosensitivity of HeLa cells to low linear energy transfer (LET) γ-irradiation and high LET carbon-ion beam irradiation, which is consistent with the recent report showing that temporal miR-145 depletion after IR contributes to radioresistancy through DNMT3B upregulation^37^. In non-irradiated cells, knockdown of *DNMT3B* itself did not alter cell cycle progression, the expression levels of genes involved with the DNA damage response, or the level of γH2AX; however, knockdown of this gene did enhance γ-irradiation-induced accumulation of cells in the S-G2/M phase through the disturbance of chromatin regulation and DNA damage responses. Importantly, knockdown of *DNMT3B* impaired the irradiation-induced formation of HP1β foci and regulation of H2AX, leading to attenuated induction of RAD51 foci. Furthermore, upregulation of *P21-CIP1*, *BAX, NOXA*, and *PUMA* could underlie the radiosensitizing effect of *DNMT3B* knockdown.

Histone H2AX is responsible for efficient DNA damage checkpoint responses through its phosphorylated form, γH2AX. H2AX knockout cells show enhanced radiosensitivity and defective DSB repair^36^,^38^,^39^. Accordingly, we confirmed that *H2AX* overexpression leads to radiosensitization effect. A recent study showed that H2AX is accumulated after DNA damage and this H2AX accumulation is significant for efficient repair^35^. However, it is not fully elucidated how this accumulation is regulated. In this study, we demonstrated that *DNMT3B* knockdown induced impairment of H2AX accumulation after γ-irradiation. Furthermore, the PLA assay suggested that DNMT3B directly or indirectly interacts with H2AX after irradiation. Since we did not detect differences in *H2AX* expression levels between *DNMT3B* RNAi and control conditions after irradiation, out data suggests that DNMT3B enhanced the stability of H2AX protein after irradiation. This could explain the low intensity of γ-H2AX in *DNMT3B* knockdown cells after irradiation. We also found that CHK2 phosphorylation levels are decreased in *DNMT3B* knockdown cells after γ-irradiation (Fig. 4C), implying that phosphorylation signalling of ATM/ATR might also be impaired and contributes to attenuated γH2AX induction. Additionally, we demonstrated that; 1) *DNMT3B* RNAi attenuated IR-dependent activation of the G1/S checkpoint, which is a similar phenotype to H2AX knockout cells, and 2) *H2AX* overexpression attenuated the radiosensitization by *DNMT3B* RNAi. These results indicate that impairment of H2AX regulation after IR induced by DNMT3B dysfunction contributes to radiosensitization .

The importance of HP1β to activation of DNA damage response has been described previously^31^,^40^,^41^. After γ-irradiation, HP1 immediately accumulates at DSB sites^32^. After IR, HP1β is initially phosphorylated and released from H3K9me2/3 regions, although it returns at a later stage^34^. The results presented here demonstrate that the irradiation-induced formation of HP1β foci is impaired when DNMT3B is dysfunctional. This process is accompanied by disappearing PLA foci between HP1β and DNMT3B, suggesting that DNMT3B did not guide HP1β to DSB sites by direct interaction. One possibility is that co-localizing DNMT3B and HP1β in heterochromatin is a basal state to release HP1β from H3K9me residues on chromatin, whereas it is also conceivable that the indirect interaction between HP1β and DNMT3B contributes to HP1β foci formation after IR.

P53 pathway has been shown to act as a major anti-cancer barrier by inducing senescence and apoptosis. It is known that P53 takes a part in ARF/MDM2/P53 module and ARF is frequently inactivated in the tumors expressing wild-type P53^42^; the presence of *P53* mutation and *P53* promoter methylation in HCT116 was identified^43^ and in A549, *P53* expression is silenced^44^. Our current study therefore demonstrated that *DNMT3B* RNAi radiosensitized p53 dysfunctional cancer cell lines, namely HeLa, A549, and HCT116.

DNMT3A and DNMT3B are overexpressed in various cancers^45^ and DNMT3B expression is essential for embryogenesis; however, its expression in adults is restricted to organs such as the testes and spleen^46^,^47^. In addition, unlike the inhibition of DNMT1, which induces DNA lesions and stalling of replication forks^48^,^49^, knockdown of *DNMT3B* did not induce γH2AX foci in the absence of irradiation. A few genes that cause radiosensitization to carbon-ion beam irradiation, including PARP and heat-shock protein 90, have been reported to date^20^. Here, knockdown of *DNMT3B* also increased the radiosensitivity of several cancer cell lines including HeLa cells to carbon-ion beam irradiation. Overall, these findings suggest that DNMT3B could be a useful target for the induction of tumor-selective radiosensitization.

Epigenetic regulators are thought to be involved in DNA damage responses mainly through transcriptional regulation of multiple DNA repair factors. DNMT1 is involved in mismatch repair and maintenance of microsatellite stability^50^. DNMT3A and DNMT3B interact with DNA glycosylases and stimulate the mismatch repair pathway^51^,^52^,^53^. DNMT3A and DNMT3B have been suggested as targets of cancer therapy because enhanced *DNMT3* expression can silence tumor suppressor genes in cancer cells^54^ and *DNMT3B* overexpression is reported as a poor prognostic factor in patients^16^. Together with accumulating evidence, the results presented here highlight the value of DNMT3 as a target of cancer therapy. Although previous reports showed that the epigenetic modifier 5-aza-2-deoxycytidine inhibits DNMT3B, its major target seems to be DNMT1^55^, and to our knowledge a specific inhibitor of DNMT3B is not currently available. Exposure of various cancer cell lines to 5-aza-2-deoxycytidine causes radiosensitization accompanied by inhibition and downregulation of DNMT1 and DNMT3^17^,^56^; therefore, further research into the development of specific inhibitors of DNMT3B is required.

In conclusion, this report describes the identification of radiosensitization targets utilizing a comprehensive genome-wide analysis, and demonstrates the effectiveness of gene cluster analyses for validation and evaluation of potential targets. Additional comprehensive analyses using different cancer cell types will enable the identification of sensitizing clusters of genes.

## Additional Information

**How to cite this article**: Fujimori, H. *et al.* A comprehensive analysis of radiosensitization targets; functional inhibition of DNA methyltransferase 3B radiosensitizes by disrupting DNA damage regulation. *Sci. Rep.* **5**, 18231; doi: 10.1038/srep18231 (2015).

## Supplementary Material

Supplementary Information
