# Supplementary material for: A comprehensive analysis of radiosensitization targets; functional inhibition of DNA methyltransferase 3B radiosensitizes by disrupting DNA damage regulation
Source: Sci Rep. 2015 Dec 15;5:18231. doi: 10.1038/srep18231 (PMC4678329; doi:10.1038/srep18231)
Supplement: Supplementary Information [file srep18231-s1.pdf]

Supplementary information of

A comprehensive analysis of radiosensitization targets; functional inhibition of DNA methyltransferase 3B radiosensitizes by disrupting DNA damage regulation

Hiroaki Fujimori[1,2], Akira Sato[1], Sota Kikuhara[1,2,3], Junhui Wang[1,4], Takahisa Hirai[1,5], Yuka Sasaki[2], Yasufumi Murakami[3], Ryuichi Okayasu[6] and Mitsuko Masutani[1,2,7]*

[1]Division of Genome Stability Research, National Cancer Center Research Institute, 5-1-1 Tsukiji, Chuo-ku, Tokyo 104-0045, Japan.
[2]Division of Chemotherapy and Translational Research, National Cancer Center Research Institute, 5-1-1 Tsukiji, Chuo-ku, Tokyo 104-0045, Japan.
[3]Department of Biological Science and Technology, Faculty of Industrial Science and Technology, Tokyo University of Science, 2641 Yamazaki, Noda, Chiba 278-8510, Japan.
[4]Department of Molecular Genetics, Medical Research Institute, Tokyo Medical and Dental University, 1-5-45 Yushima, Bunkyo-ku, Tokyo 103-8501, Japan.
[5]Department of Radiation Oncology, Juntendo University Faculty of Medicine, 2-1-1 Hongo, Bunkyo-ku, Tokyo 113-8421, Japan.
[6]Open Laboratory/Research Center for Radiation Protection, National Institute of Radiological Sciences, 4-9-1 Anagawa, Inage, Chiba 263-8555, Japan.
[7]Department of Frontier Life Sciences, Nagasaki University Graduate School of Biomedical Sciences, 1-7-1, Sakamoto, Nagasaki 852-8588, Japan

***Corresponding author**: Mitsuko Masutani, Tel: +81-95-819-8502, Fax: +81-95-819-8502, E-mail: mmasutan@nagasaki-u.ac.jp

Running title: Radiosensitization target screening and evaluation of DNMT3B

Supplemental Fig. 1:

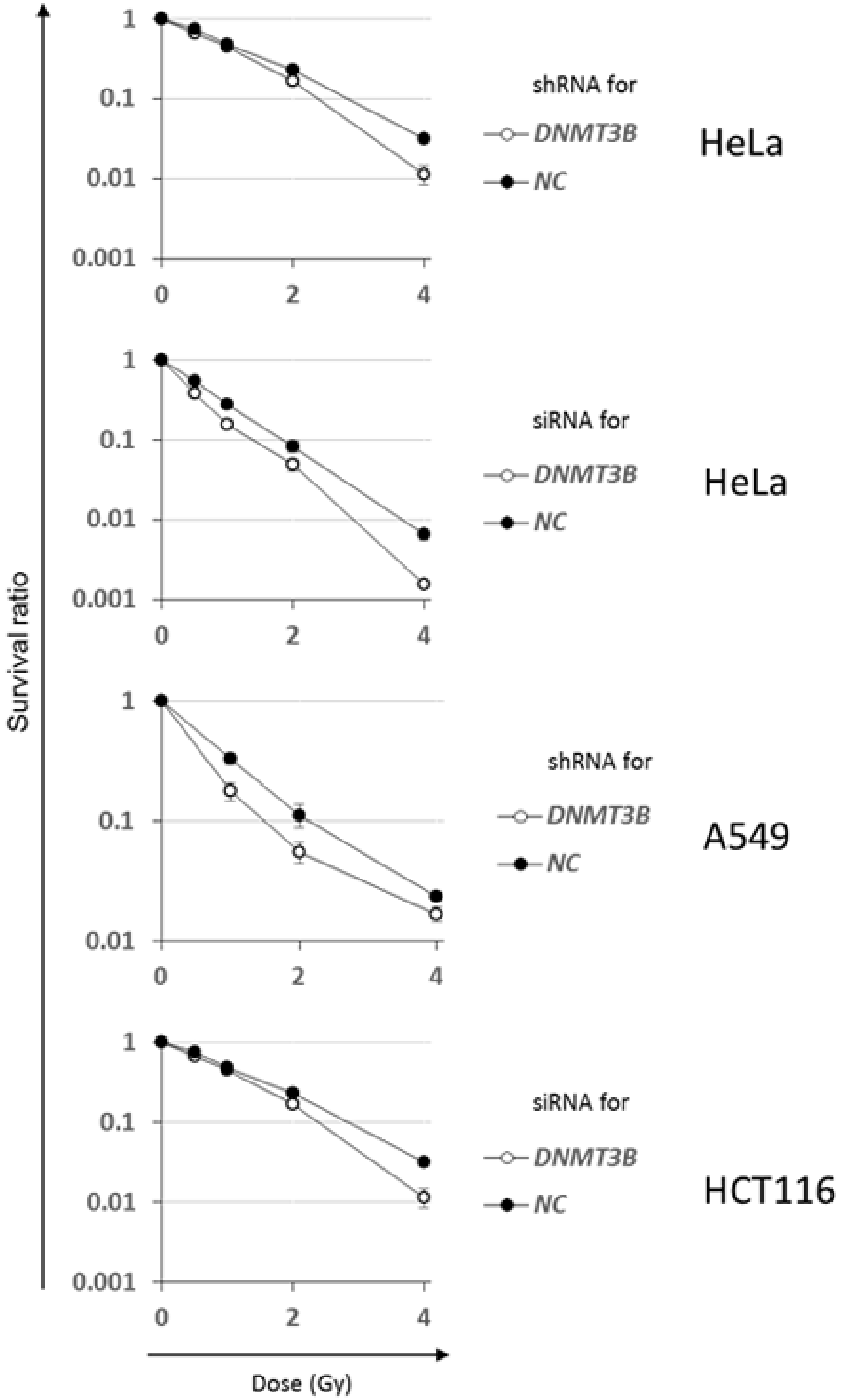

Supplemental Fig. 2:

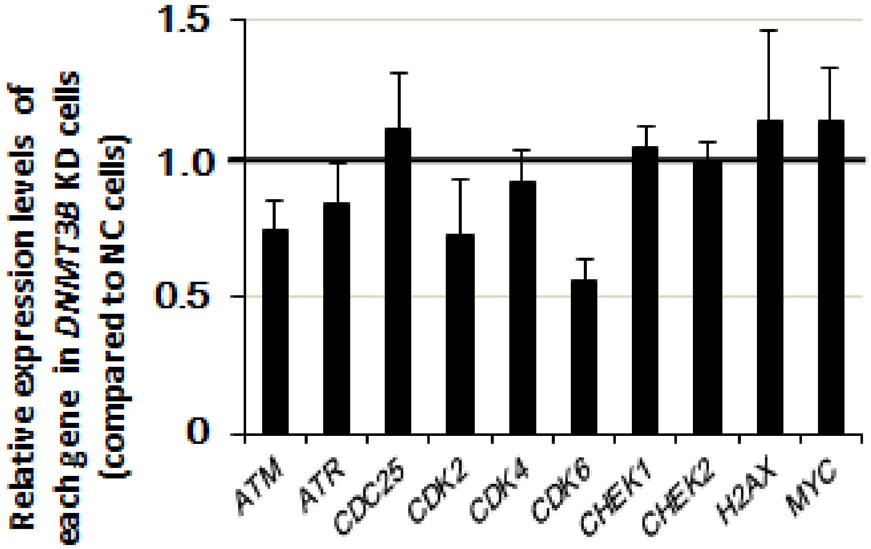

Supplemental Fig. 3:

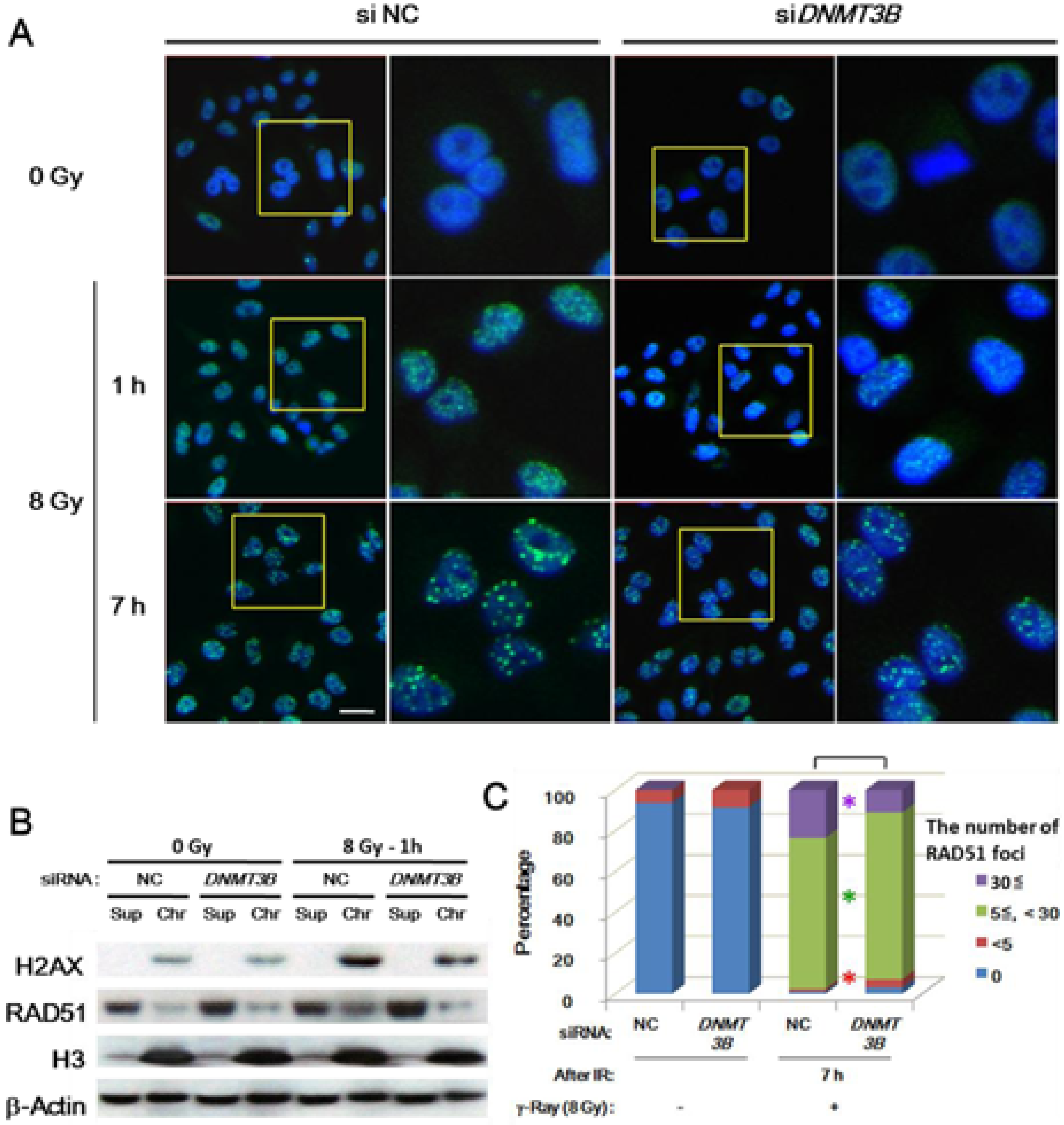

Supplemental Fig. 4:

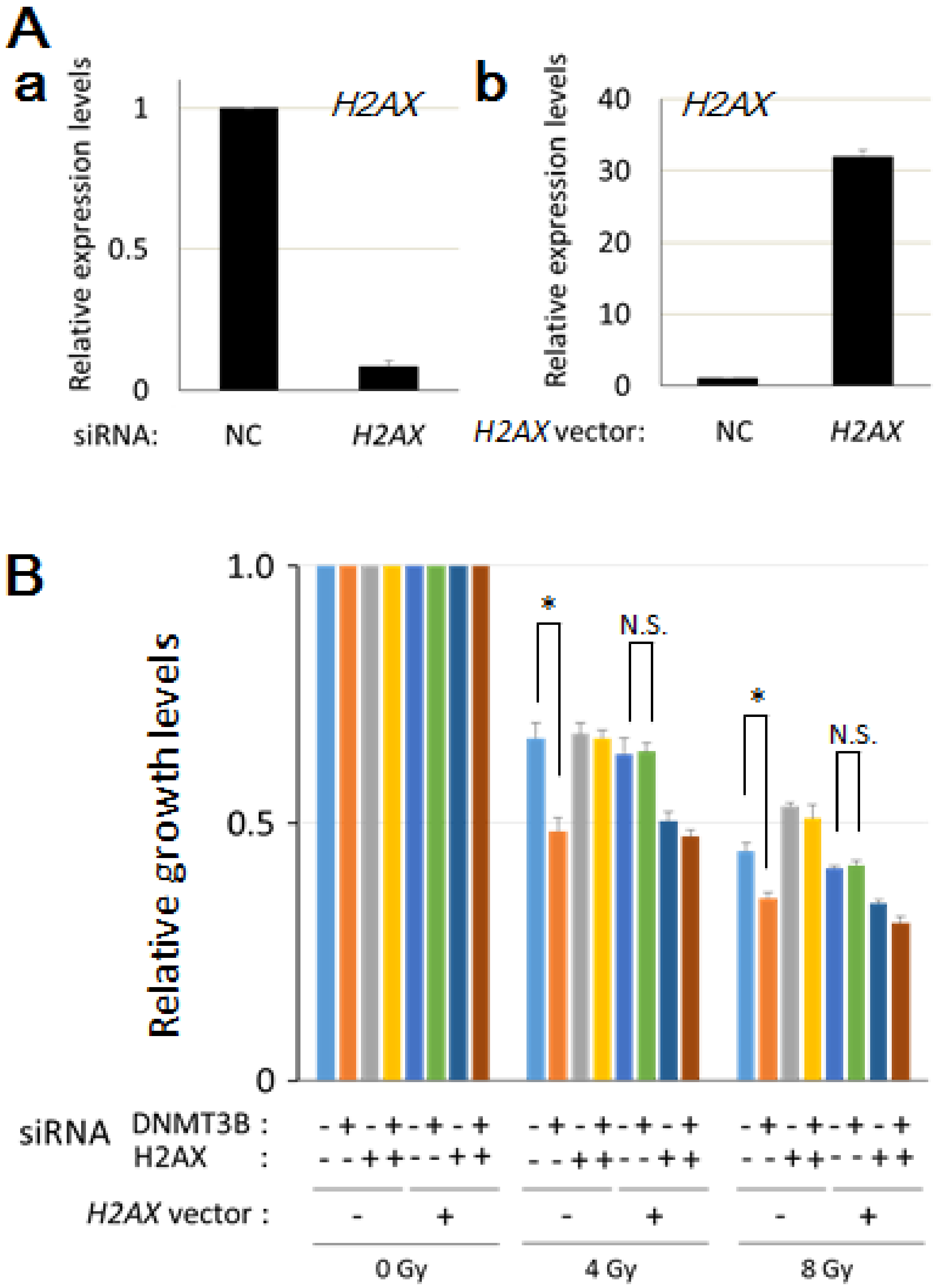

Supplemental figure legends

Figure S1. Radiosensitization by combination of *DNMT3B* RNAi and carbon-ion beam irradiation in *DNMT3B* expressing cancer cell lines.

The enhancement ratios at 10% survival were 1.4 (shRNA in HeLa), 1.3 (siRNA in HeLa), 1.6 (shRNA in A549) and 1.7 (siRNA in HCT116). Error bars: SE. Asterisks show $P<0.05$.

Figure S2. Gene expression alteration by *DNMT3B* RNAi in HeLa cells.

RNAi for *DNMT3B* did not induce major alteration of gene expression levels for DSB sensor (*ATM*, *ATR* and *H2AX*), checkpoint (*CDC25*, *CDK2*, *CDK4*, *CDK8*, *CHEK1* and *CHEK2*) and cell cycle accelerator (*MYC*) in HeLa cells. The expression levels were normalized to that of *GUSB*. Error bars: SE.

Figure S3. Knockdown of *DNMT3B* knockdown mitigates the recruitment of RAD51 to chromatin after γ-irradiation.

(A) The formation of RAD51 foci 1 h after γ-irradiation of control and *DNMT3B* knockdown cells. (B) Recruitment of RAD51 to chromatin fraction 1 h after γ-irradiation of control and *DNMT3B* knockdown cells. Sup: The nucleoplasm obtained as supernatant fraction. (C) The formation of RAD51 foci 7 h after γ-irradiation of control and *DNMT3B* knockdown cells. The RAD51 foci were counted at least in 141 cells, respectively. Scale bar: 20 μm. Asterisks show $P<0.05$ (χ2-test).

Figure S4. Gene expression alteration by *DNMT3B* RNAi in HeLa cells.

(A) Relative expression levels of *H2AX* in HeLa cells transfected *H2AX* siRNA (a) or *H2AX* overexpression vector (b). The expression level was normalized to that of *GUSB*. Error bars: SE. (B) Growth levels of HeLa cells in each transfected conditions. As in figure 6Ab, each growth radio was also normalized to correspondent control condition (transfected with negative-control siRNA and control vector).

Supplemental Table 1 List of PCR primers

Genes	Forward primers (5'-3')	Reverse primers (5'-3')
**qRT-PCR***		
ATM	GACAGCTTGTTAAGGGCCGTG	GCTGAGAGAGGGGAACCAC
ATR	TGAGGGATGCCACTGCTTGT	TGTCCACTCGGACCTGTTAGC
BAX	AACATGGAGCTGCAGAGGATGATT	TCTGATCAGTTCCGGCACCTT
CDC25	TTTCCAAGGTATGTGCGCTGC	GACCCTGGAACTTCCCCGAC
CDK2	CCCATGAGGTGGTGACCCTGTG	GAATAGGGCCCGGCGAGTC
CDK4	TCTGGTACCGAGCTCCCGAA	GAGGCAGCCCAATCAGGTCAA
CDK6	GGTCGTCACGCTGTGGTACA	TGGGAGTCCAATCACGTCCA
CHEK1	TGGGACCAACCCAGTGACAG	TTTGCCCCTTTCTTGAGGGGT
CHEK2	CCCCTGGGCTCGATTATGGG	ACCCACTTCCCTGAAAATCCGA
CUL1	GCGGGTTCGAGTACACCTCT	ACGTTCCAACTCTGACGGCAA
DNMT3B	AATCCTGGAGGCTATCCGCAC	CGCCTGTCAAGTCCTGTGTGT
DUSP22	GGCCTGTACATCGGCAACTT	GCACAGGTATTTAACTCCCTCCA
FBXW7	ACGGGGGCACAGAATCACTG	AAGAGCGGACCTCAGAACCA
GUSB	GCCTGCGTCCCACCTAGAAT	ACATACGGAGCCCCCTTGTC
H2AX	GCCATCCGCAACGACGACGAGG	CTTAGTACTCCTGGGAGGCCTGG
KRAS	CTGGTGGCGTAGGCAAGAGT	CCTCTTGACCTGCTGTGTCG
MYC	GGGGAGGCTATTCTGCCCAT	GCTAACGTTGAGGGGCATCG
NOXA	AAGAACGCTCAACCGAGCCC	GAAGGAGTCCCCTCATGCAAGT

P16-INK4A	TGTCCTTGGGCTGCCTGTTTT	GCCATTAGCGCATCACAGTCG
P21-CIP1	GCATAGAACAGGCTGGTGGC	GGTTCTGACGGACATCCCCA
TP63A	ACCAGAGATGGGCAAGTCCTG	AAACGGGCGCTTCGTACCAT
PUMA	GGACGACCTCAACGCACAG	ATTGGGCTCCATCTCGGGGG
YES1	AGCTGCACTGTATGGTCGGT	CACGGTTCACCATACCTGGA
**Cloning****		
DNMT3B	TGGCGGCCGCCCACCATGAAGGGAGACACC	GAGTCGACGTTCACATGCAAAGTAG
H2AX	TGGCGGCCGCCCACCATGTCGGGCCGCGGC	GAGTCGACGGTACTCCTGGGAGGCC

* qRT-PCR was performed in 2 step PCR condition as 94°C 10 sec-62°C 40 sec.

**ORF sequences of *DNMT3B* and *H2AX* were amplified by 3 step PCR condition as 98 ^{o}C 10 sec-58 ^{o}C 15 sec-72 ^{o}C 15 or 50 sec, respectively.

Supplemental Table 2 List of dsOligos for BLOCK-iT inducible Pol II miR RNAi Expression vector

Genes	Top strand sequences (5'-3')
FBXW7	TGCTGTAATAGCTCTGTTCCAAGGAAGTTTTGGCCACTGACTGACTTCCTTGGCAGAGCTATTA
CUL1	TGCTGAGTGCTGCCAACGTGACAAGTGTTTTGGCCACTGACTGACACTTGTCATTGGCAGCACT
DNMT3B	TGCTGTATCTAAGCTCCTTGCTTCACGTTTTGGCCACTGACTGACGTGAAGCAGAGCTTAGATA
KRAS	TGCTGAATGCATGACAACACTGGATGGTTTTGGCCACTGACTGACCATCCAGTTGTCATGCATT
YES1	TGCTGTAAATAAGCAGGAGCCTCACTGTTTTGGCCACTGACTGACAGTGAGGCCTGCTTATTTA

Supplemental Table 3 List of antibodies

Primary antibodies	Dilution	Maker	Secondary antibodies	Dilution	Maker
**Western blot**					
Anti-phospho-Histone H2A.X (Ser139), (JBW301)	2,0000	Merck-Millipore (Darmstadt, Germany)	Goat anti-mouse IgG-HRP	20,000	Beckman (Fullerton, CA)
Monoclonal Anti-β-Actin antibody produced in mouse, (AC74)	2,0000	Sigma (St. Louis, MO)	Goat anti-mouse IgG -HRP	20,000	Beckman
Anti-H2AX Antibody, Affinity Purified	2,0000	Bethyl (Montgomery, TX)	Goat anti-rabbit IgG -HRP	20,000	BioRad (Hercules, CA)
Anti-DNMT3B	1,000	Novous (Littleton, CO)	Goat anti-rabbit IgG -HRP	30,000	Beckman
Anti- Phospho-Chk2 (Thr68) (C13C1)	1,000	Cell Signaling Technology (Danvers, MA)	Goat anti-mouse IgG -HRP	30,000	Beckman
Anti-phospho-Akt XP (Ser473) (D9E)	2,000	Cell Signaling Technology	Goat anti-rabbit IgG -HRP	30,000	Beckman
Anti-histone H3 XP (D1H2)	1,000	Cell Signaling Technology	Goat anti-rabbit IgG -HRP	30,000	Beckman
Anti-Rad51(human) antibody	4,000	Bio Academia (Osaka, Japan)	Goat anti-rabbit IgG -HRP	30,000	Beckman
DYKDDDDK Tag (9A3) mouse mAb	1,000	Cell Signaling Technology	Goat anti-mouse IgG -HRP	30,000	Beckman
**Immunocytochemistry**					
Anti-phospho-Histone H2A.X (Ser139), (JBW301)	500	Merck-Millipore	Goat anti-mouse IgG Alexa Fluor 488 conjugate	1,000	Life Technologies
Anti-Rad51(human) antibody	500	Bio Academia	Goat anti-rabbit IgG	1,000	Life

			Alexa Fluor 488 conjugate		Technologies
Anti-HP1β XP (D2F2)	800	Cell Signaling Technology	Goat anti-rabbit IgG Alexa Fluor 594 conjugate	1,000	Life Technologies
Di/Tri-Methyl-Histone H3 (Lys9) (6F12)	100	Cell Signaling Technology	Goat anti-rabbit IgG Alexa Fluor 488 conjugate	1,000	Life Technologies
**Duolink in situ PLA**					
Anti-HP1β XP (D2F2)	500	Cell Signaling Technology	Duolink® In Situ PLA® Probe Anti-Rabbit PLUS	5	Sigma
Anti-H2AX Antibody, Affinity Purified	500	Bethyl	Duolink® In Situ PLA® Probe Anti-Rabbit PLUS	5	Sigma
DYKDDDDK Tag (9A3) mouse mAb	500	Cell Signaling Technology	Duolink® In Situ PLA® Probe Anti-Mouse MINUS	5	Sigma
